# Bibliometric analysis of electroencephalogram research in Parkinson’s disease from 2004 to 2023

**DOI:** 10.3389/fnins.2024.1433583

**Published:** 2024-07-19

**Authors:** Xiao-Yu Liao, Ya-Xin Gao, Ting-Ting Qian, Lu-Han Zhou, Li-Qin Li, Yan Gong, Tian-Fen Ye

**Affiliations:** ^1^Department of Rehabilitation Medicine, The Affiliated Suzhou Hospital of Nanjing Medical University, Suzhou, China; ^2^The Fourth Rehabilitation Hospital of Shanghai, Shanghai, China

**Keywords:** bibliometrix, CiteSpace, VOSviewer, Parkinson’ disease, electroencephalogram, neural oscillations, visualization analysis

## Abstract

**Background:**

Parkinson’s disease (PD) is a prevalent neurodegenerative disorder affecting millions globally. It encompasses both motor and non-motor symptoms, with a notable impact on patients’ quality of life. Electroencephalogram (EEG) is a non-invasive tool that is increasingly utilized to investigate neural mechanisms in PD, identify early diagnostic markers, and assess therapeutic responses.

**Methods:**

The data were sourced from the Science Citation Index Expanded within the Web of Science Core Collection database, focusing on publications related to EEG research in PD from 2004 to 2023. A comprehensive bibliometric analysis was conducted using CiteSpace and VOSviewer software. The analysis began with an evaluation of the selected publications, identifying leading countries, institutions, authors, and journals, as well as co-cited references, to summarize the current state of EEG research in PD. Keywords are employed to identify research topics that are currently of interest in this field through the analysis of high-frequency keyword co-occurrence and cluster analysis. Finally, burst keywords were identified to uncover emerging trends and research frontiers in the field, highlighting shifts in interest and identifying future research directions.

**Results:**

A total of 1,559 publications on EEG research in PD were identified. The United States, Germany, and England have made notable contributions to the field. The University of London is the leading institution in terms of publication output, with the University of California closely following. The most prolific authors are Brown P, Fuhr P, and Stam C In terms of total citations and per-article citations, Stam C has the highest number of citations, while Brown P has the highest H-index. In terms of the total number of publications, Clinical Neurophysiology is the leading journal, while Brain is the most highly cited. The most frequently cited articles pertain to software toolboxes for EEG analysis, neural oscillations, and PD pathophysiology. Through analyzing the keywords, four research hotspots were identified: research on the neural oscillations and connectivity, research on the innovations in EEG Analysis, impact of therapies on EEG, and research on cognitive and emotional assessments.

**Conclusion:**

This bibliometric analysis demonstrates a growing global interest in EEG research in PD. The investigation of neural oscillations and connectivity remains a primary focus of research. The application of machine learning, deep learning, and task analysis techniques offers promising avenues for future research in EEG and PD, suggesting the potential for advancements in this field. This study offers valuable insights into the major research trends, influential contributors, and evolving themes in this field, providing a roadmap for future exploration.

## 1 Introduction

Parkinson’s disease (PD) is the second most common central neurodegenerative disorder globally. It primarily affects the nigrostriatal dopaminergic system and is accompanied by degeneration of other non-dopaminergic neural circuits (Hayes, 2019). The primary clinical manifestations of this disease encompass both motor and non-motor symptoms, which significantly impact the quality of life and survival time of patients (Reich and Savitt, 2019). The diagnosis of dyskinesia is primarily based on clinical assessment, which involves the identification of characteristic motor symptoms such as bradykinesia, tremor, rigidity, postural instability, and gait disturbances (Bloem et al., 2021). The integration of genetic markers with imaging techniques, such as MRI and DAT-SPECT, facilitates the differentiation of PD from other comparable movement disorders. Moreover, non-motor symptoms like decreased olfaction and rapid eye movement sleep behavior disorder present diagnostic value in early-stage PD. Electroencephalography (EEG) is a pivotal non-invasive methodology for evaluating cerebral activity with high temporal resolution, enabling the capture of rapid dynamic alterations in cerebral processes (Waninger et al., 2020). It is particularly suitable for studying neural oscillations, brain network connections, and the effects of various stimuli or cognitive tasks on brain function. In addition, in comparison to MRI and DAT-SPECT, EEG is relatively cost-effective, straightforward to utilize, and applicable to a diverse range of clinical and research settings. These properties render EEG an invaluable tool for basic neuroscience research and clinical diagnosis. In PD, motor control is critically dependent on the synchronization and functional connectivity among cortical regions, which are substantially influenced by the dynamics within the nigrostriatal-thalamic circuitry (Lalo et al., 2008). This implies that EEG can be employed as a non-invasive and dynamic instrument for the detection of synchronization and functional connectivity between cortical areas in PD patients, with the objective of monitoring treatment response and potential disease progression. Bibliometrics analysis represents a novel approach to research outcome statistics, furnishing researchers with qualitative and quantitative insights into literature characteristics through the analysis of variables including countries, institutions, authors, journals, references and keywords (Durieux and Gevenois, 2010). The predominant bibliometric software, such as VOSviewer and CiteSpace, facilitates the visualization of extensive publication datasets. Bibliometric analysis also helps to identify leading authors, prolific research institutions, and influential and high-quality publications, which can be used to develop guidelines, pinpoint research hotspots, and predict research trends (Chen et al., 2024). To date, bibliometric studies focusing on EEG in PD have not been reported in the literature. Consequently, this research employs bibliometric analysis tools to delineate and track the evolution of research hotspots in this field from 2004 to 2023. Furthermore, it outlines prospective avenues for future investigation.

## 2 Materials and methods

### 2.1 Data source and collection

**Figure 1:**
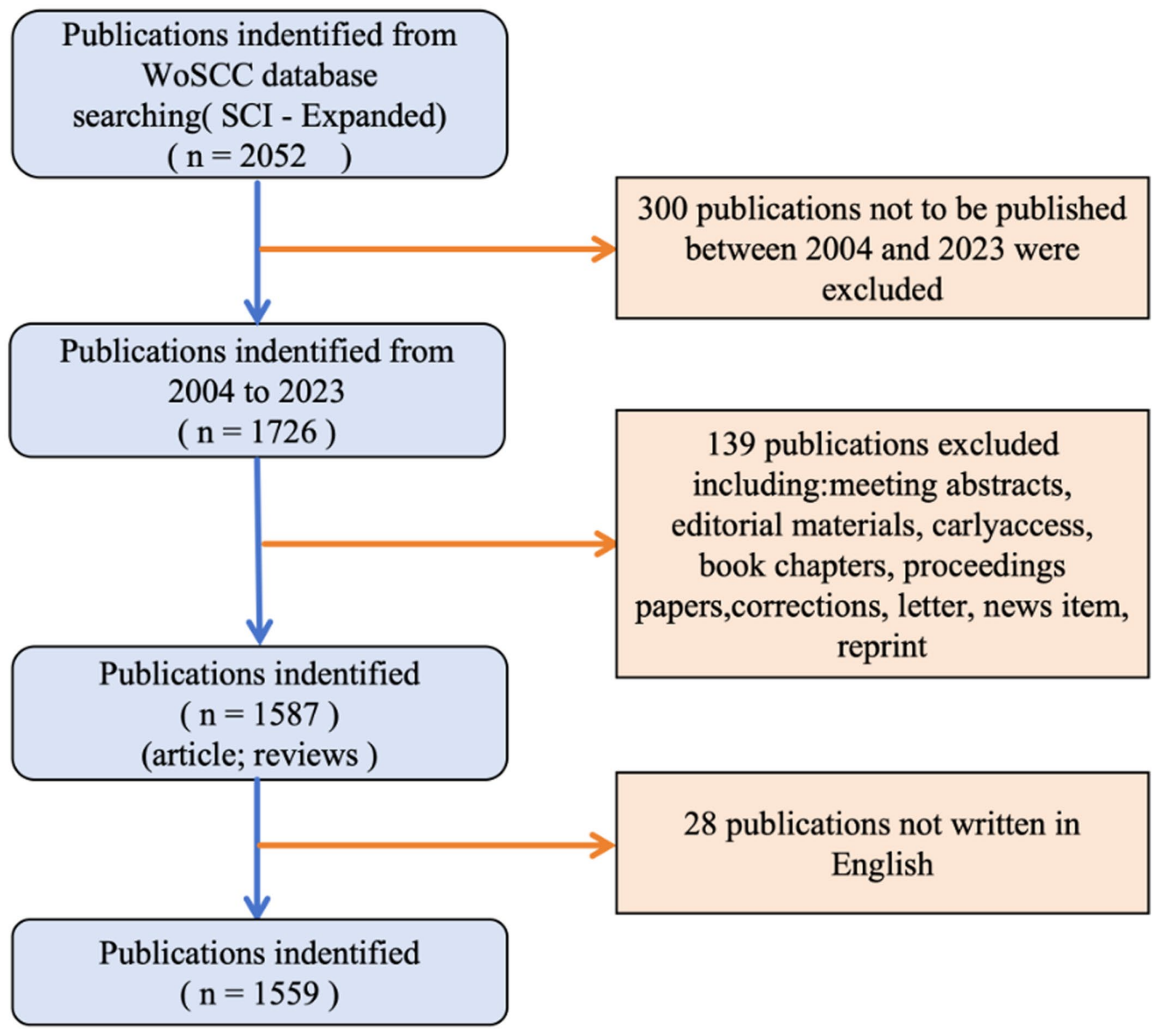
Flow chart of literature screening.

The data for the bibliometric analysis were obtained from the Science Citation Index Expanded (SCI-Expanded) within the Web of Science Core Collection database (WoSCC). The data retrieval strategy was summarized as follows: #1:TS = (“Parkinson Disease” OR Parkinson* OR “Idiopathic Parkinson’s Disease” OR “Lewy Body Parkinson’s Disease” OR “Parkinson’s Disease, Lewy Body” OR “Parkinson Disease, Idiopathic” OR “Parkinson’s Disease, Idiopathic” OR “Idiopathic Parkinson Disease” OR “Lewy Body Parkinson Disease” OR “Parkinsonism, Primary” OR “Primary Parkinsonism” OR “Paralysis Agitans”); #2:TS = (Electroencephalography OR EEG OR Electroencephalogram∗); the ultimate dataset: #1 AND #2. The use of a truncation symbol “∗” prevented missed detections and improved retrieval effects. The study included only English-language studies. The time of search period was between 1 January 2004 and 31 December 2023. The search strategy is depicted in Figure 1. In order to minimize the potential for bias resulting from routine database updates, the literature search was conducted on a specific date. A total of 1,559 documents were retrieved in the search, including reviews and articles. Upon completion of the retrieval process, the data were saved as complete records and cited references. Articles were extracted and exported in either “Plain text file” or “Tab-delimited file” formats.

### 2.2 Bibliometric analysis

The literature that met the inclusion criteria was exported as a plain text file named “download_xxx.txt,” which comprised full records and cited references. Subsequently, the file was imported into VOSviewer 1.6.19 and CiteSpace 6.2.R2 software in order to construct knowledge maps and perform statistical analysis. Concurrently, the documents were exported in tab-delimited file formats and uploaded to an online analysis platform for document metrology, with the objective of generating a knowledge map of national/regional collaborative networks. VOSviewer software was employed with the following parameter settings: association strength was selected as the normalization method, and minimum thresholds for country/region, institution, and author were set at 5, 10, and 6, respectively, based on publication counts. Additionally, the minimum thresholds of 100 citations were set for authors, journals, and literature. Furthermore, the frequency of occurrence for keywords was considered, with a minimum threshold of 10. The CiteSpace software was configured with the following parameter settings: the time span was set from January 2004 to December 2023, with a time slice of 1 year. Node types were set to include keyword and reference, and the selection criteria were established to identify the g-index *k* = 25 for each slice. The pruning options employed included pathfinder, sliced networks pruning, and merged network pruning, while all other settings were retained at their default values. In this study, we initially analyze the quantity of literature, countries, institutions, authors, journals and co-cited references to summarize the current research status of EEG in PD. Subsequently, we employ co-occurrence and cluster map of keywords to identify research hotspots. Finally, we explore research frontiers and trends in this field by examining burst keywords.

## 3 Results

### 3.1 Annual publications and citations

**Figure 2:**
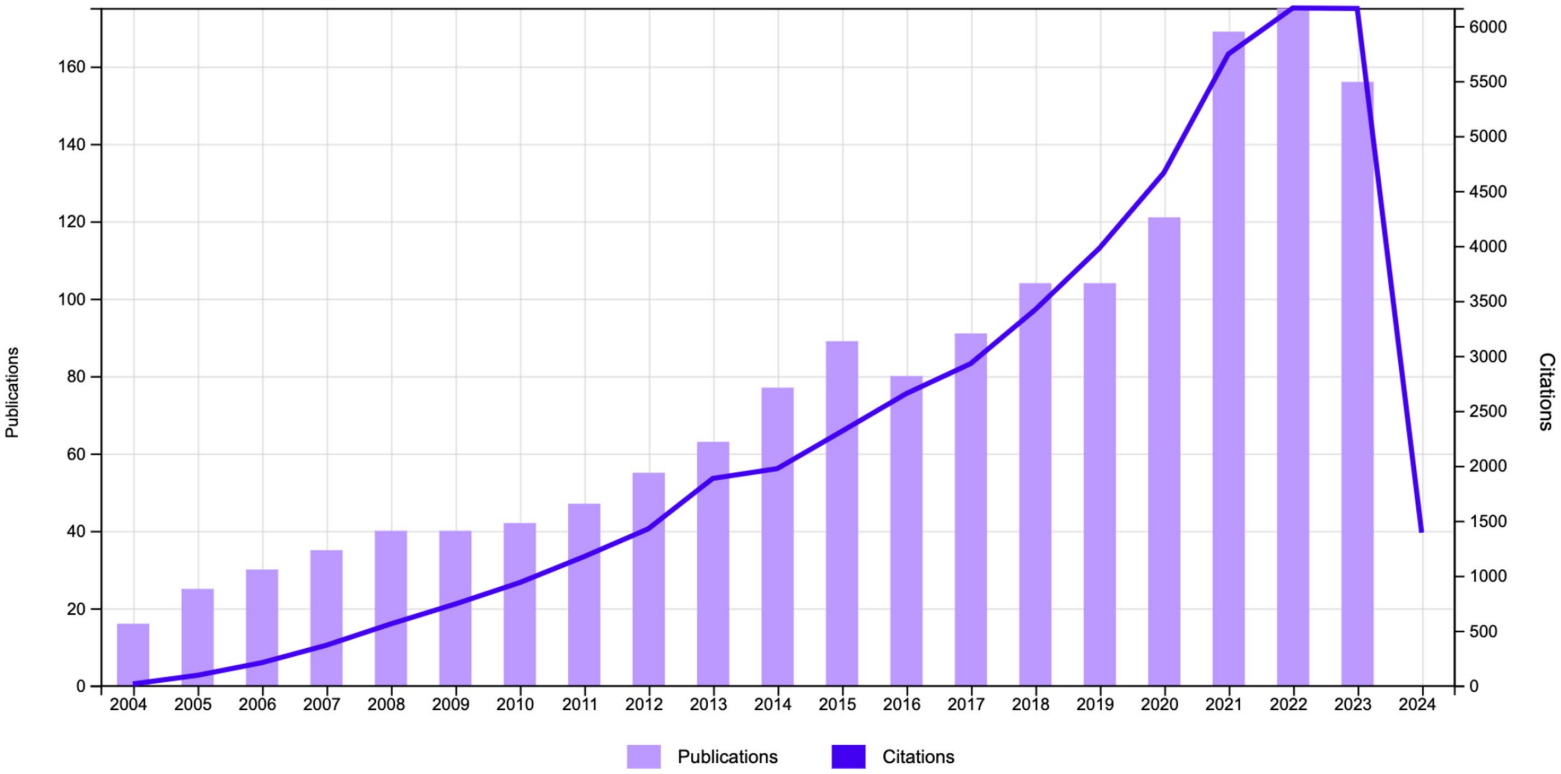
Annual publications and citations trend chart.

The study encompassed 1,559 related publications, comprising 1,354 original research articles (87%) and 205 review articles (13%). The total citations (TC) were 33,318. The average citation per publication (ACPP) was 31.25, and the h-index was 96. The dual-axis trend analysis in Figure 2 spanned 20 years, from 2004 to 2023, with time depicted on the horizontal axis. The left vertical axis denotes the number of publications, while the right vertical axis corresponds to the citation frequency. The bar graph in Figure 2 depicts the annual publication volume, which exhibits a clear upward trend over the period. The number of publications increased from 16 in 2004 to 175 in 2022, representing an almost elevenfold increase. The considerable expansion in the volume of publications is indicative of the sustained growth in interest and scholarly output in this field. The line graph depicted the citation frequency, rising from 12 in 2004 to 6,170 by 2023, signifying a remarkable increase of over 500 times. The upward trend in citation frequency became particularly evident around 2018, suggesting a significant period of growth. The interaction between the two metrics within the chart reflects the symbiotic relationship between the research output and its subsequent impact. Initially, the growth in both the number of publications and the frequency of citations was gradual, indicating a process of gradual accumulation and recognition of knowledge within the domain. The recent surge in citation numbers may reflect pioneering research breakthroughs gaining traction, indicating an increasing integration into the broader scientific discourse. Alternatively, it may signify the maturation of the domain, with earlier research findings being expanded upon with renewed vigor.

### 3.2 Distribution of countries/regions and institutions

**Figure 3:**
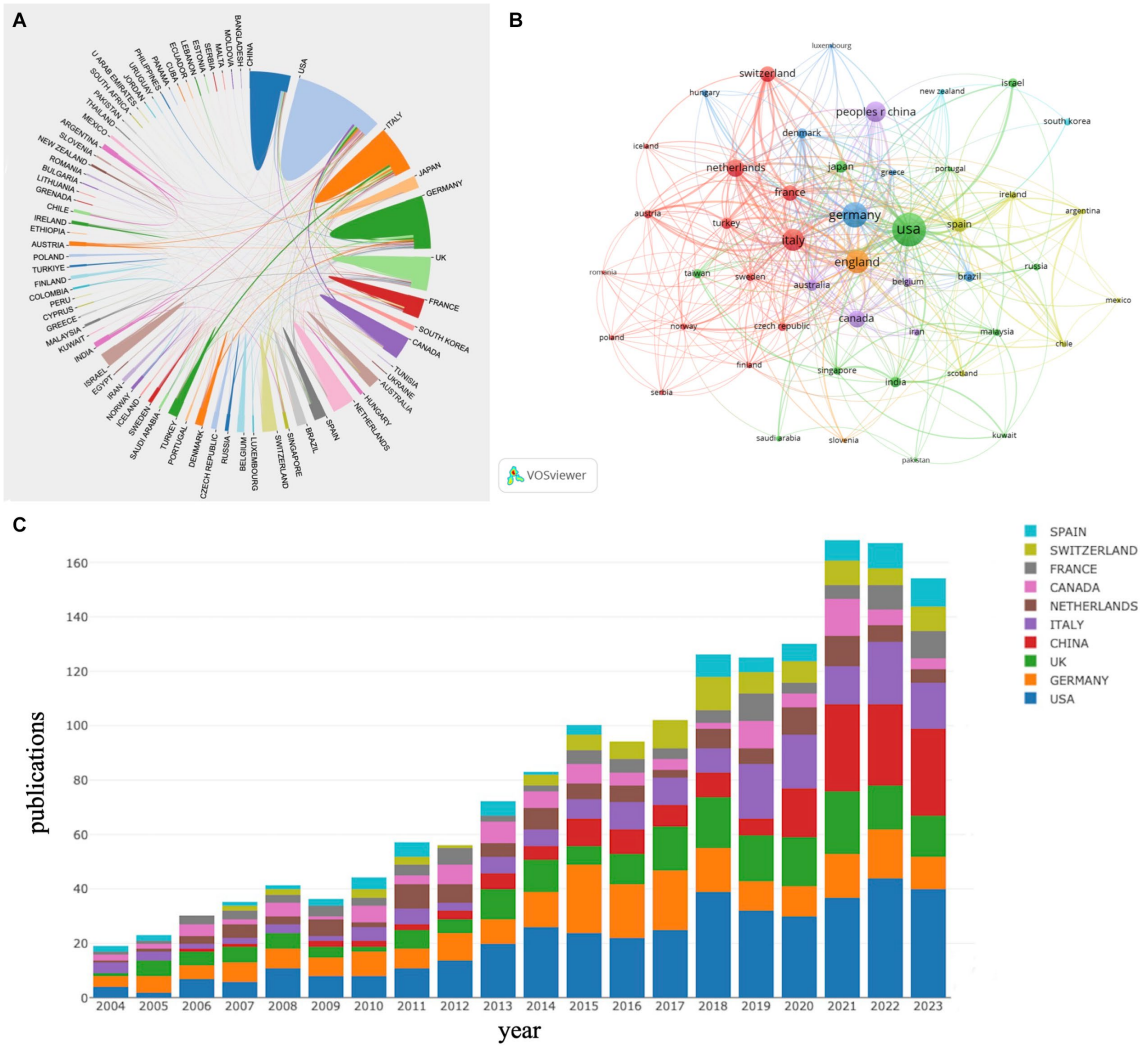
**(A)** National/regional collaborative network knowledge map. **(B)** Temporal map of national/regional cooperation network. **(C)** Stack bar plot of top 10 countries/regions in publication.

This study examined 1,559 articles produced by 2,114 institutions across 75 different countries. Among these, 218 institutions and 50 countries had published more than 5 publications in this field. As illustrated in Table 1, the United States, Germany, England, and Italy are the primary contributors of research, with the United States accounting for 26.62% (415 publications), Germany for 15.19% (412 publications), England for 12.44% (194 publications), and Italy for 11.15% (174 publications). The United States also leads in TC, h-index, and centrality, which serves to underscore its clear leadership in this field. It is noteworthy that the Netherlands has the highest ACPP despite not ranking in the top 5 for publication volume, which signifies its academic influence and high research quality. Figure 3A maps the global collaboration networks between different countries and regions, with different colored blocks representing each country or region. The size of the blocks reflects the number of articles published, while the connecting lines represent the strength of collaboration. Thicker lines indicate a stronger relationship. The United States, England, and Italy have actively collaborated with other countries, forming the core of the global research network (Lyu et al., 2024). Figure 3B illustrates that each node represents a country or region, and the lines between the nodes indicate the presence of a cooperative relationship. The thickness of the lines reflects the closeness of the collaboration. Nodes that are blue-purple indicate an earlier publication date, while yellow-green indicates a more recent publication date. Figure 3C shows that in the early timeline, a few dominant countries, represented by a limited variety of colors, contributed the majority of publications (Zhao et al., 2023). Over time, the bars have become more diverse and evenly distributed among nations, indicating a significant increase in global participation. By 2021, China had ascended to second place behind the United States in publication count, demonstrating a substantial increase in research investment and output in recent years.

**Table 1:** Top 10 countries/regions ranked by number of publications.

Rank	Countries/regions	Publications (%)	TC	ACPP	H-index	Centrality
1	United States	415(26.62%)	15,095	36.37	61	0.49
2	Germany	237(15.19%)	10,140	42.78	47	0.19
3	England	194(12.44%)	10,203	52.59	52	0.27
4	Italy	174(11.15%)	5,494	31.57	36	0.12
5	China	147(9.42%)	2,522	17.16	28	0.09
6	Netherlands	111(7.12%)	7,476	67.35	39	0.06
7	Canada	103(6.6%)	3,983	38.67	36	0.1
8	France	91(5.83%)	3,951	43.42	30	0.14
9	Switzerland	89(5.71%)	2,093	23.52	26	0.02
10	Spain	72(4.62%)	2,082	28.92	24	0.06

**Figure 4:**
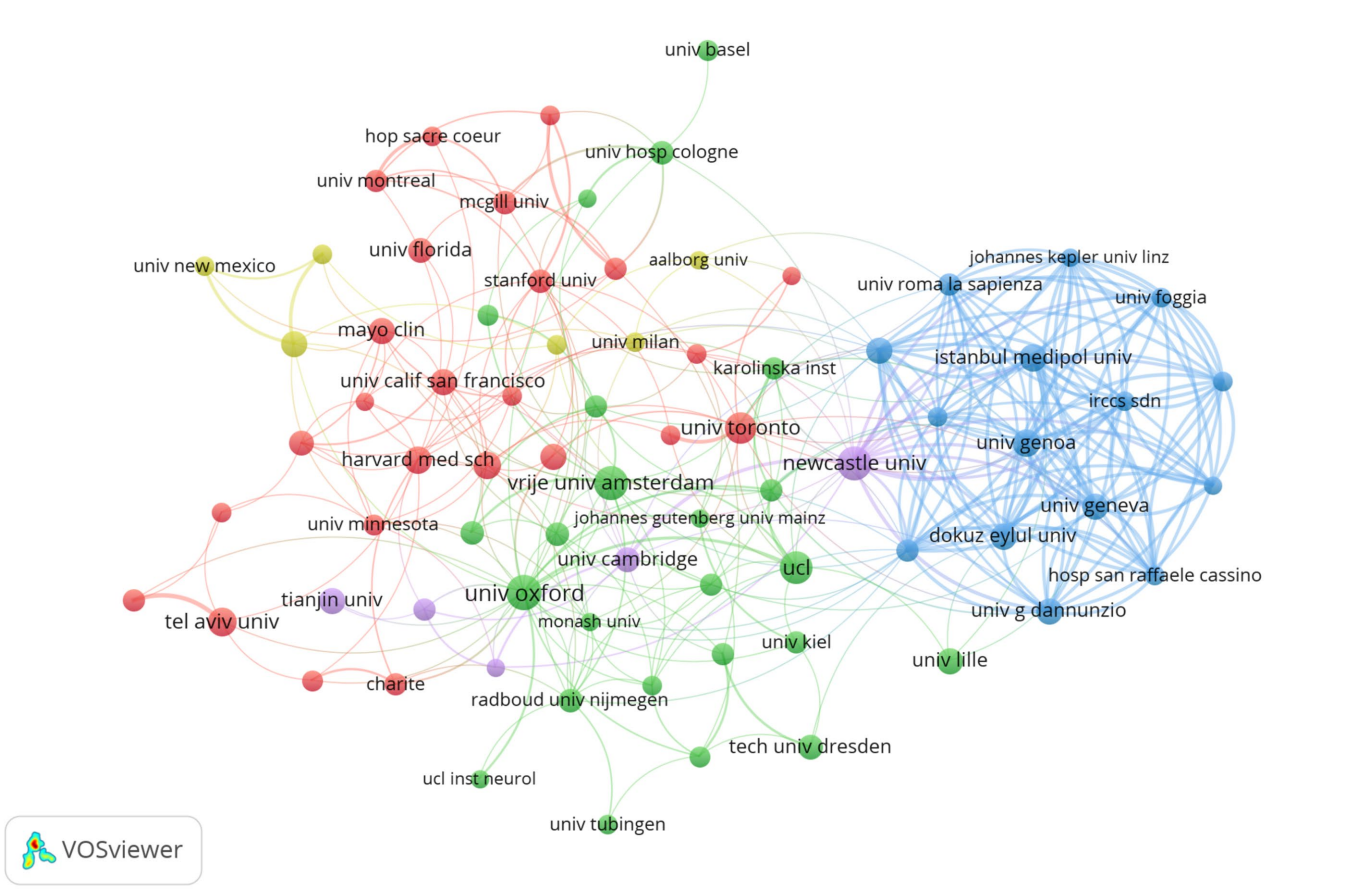
Collaborative network knowledge map of institution.

Figure 4 presents the cooperation visualization network map of institutions, created using VOSviewer, depicting collaborative relationships among institutions that have published over 10 publications in the field. The network comprises a total of 75 institutions, which have been categorized into five clusters based on the extent of their collaboration. Table 2 presents a summary of the top 10 institutions based on the number of publications. A total of 472 articles were published by these institutions, representing 30.28% of the total number of documents included in the study. In terms of the total number of publications, the University of London and the University of California have the highest number of publications. With regard to TC, the University of London is at the vanguard of this field with the highest TC (6,216), followed by the Vrije University Amsterdam in second place with TC (5,028). Among the top 10 institutions, three are from England, while the remainder are situated in the US, France, the Netherlands, Germany, and Canada. It is notable that the Vrije University Amsterdam has the highest ACPP, ranking second only to the University of London in total citations and h-index.

**Table 2:** Top 10 institutions ranked by number of publications.

Rank	Institutions	Publications (%)	TC	ACPP	H-index	Centrality	Location
1	University of London	70(4.49%)	6,216	88.8	32	0.14	England
2	University of California System	65(4.17%)	2,336	35.94	28	0.17	United States
3	University College London	57(3.65%)	4,344	76.21	29	0.31	England
4	Institut National De La Santé Et De La Recherche Medicale	51(3.27%)	1,281	25.12	17	0.1	France
5	Vrije University Amsterdam	51(3.27%)	5,028	98.59	31	0.02	Netherlands
6	University of Oxford	40(2.56%)	2,301	57.53	24	0.02	England
7	Harvard University	36(2.31%)	972	27	17	0.03	United States
8	Humboldt University of Berlin	35(2.24%)	1,866	53.31	16	0.27	Germany
9	Helmholtz Association	34(2.18%)	1,093	32.15	18	0.03	Germany
10	University of Toronto	33(2.12%)	1,463	44.33	20	0.02	Canada

### 3.3 Analysis of authors

**Figure 5:**
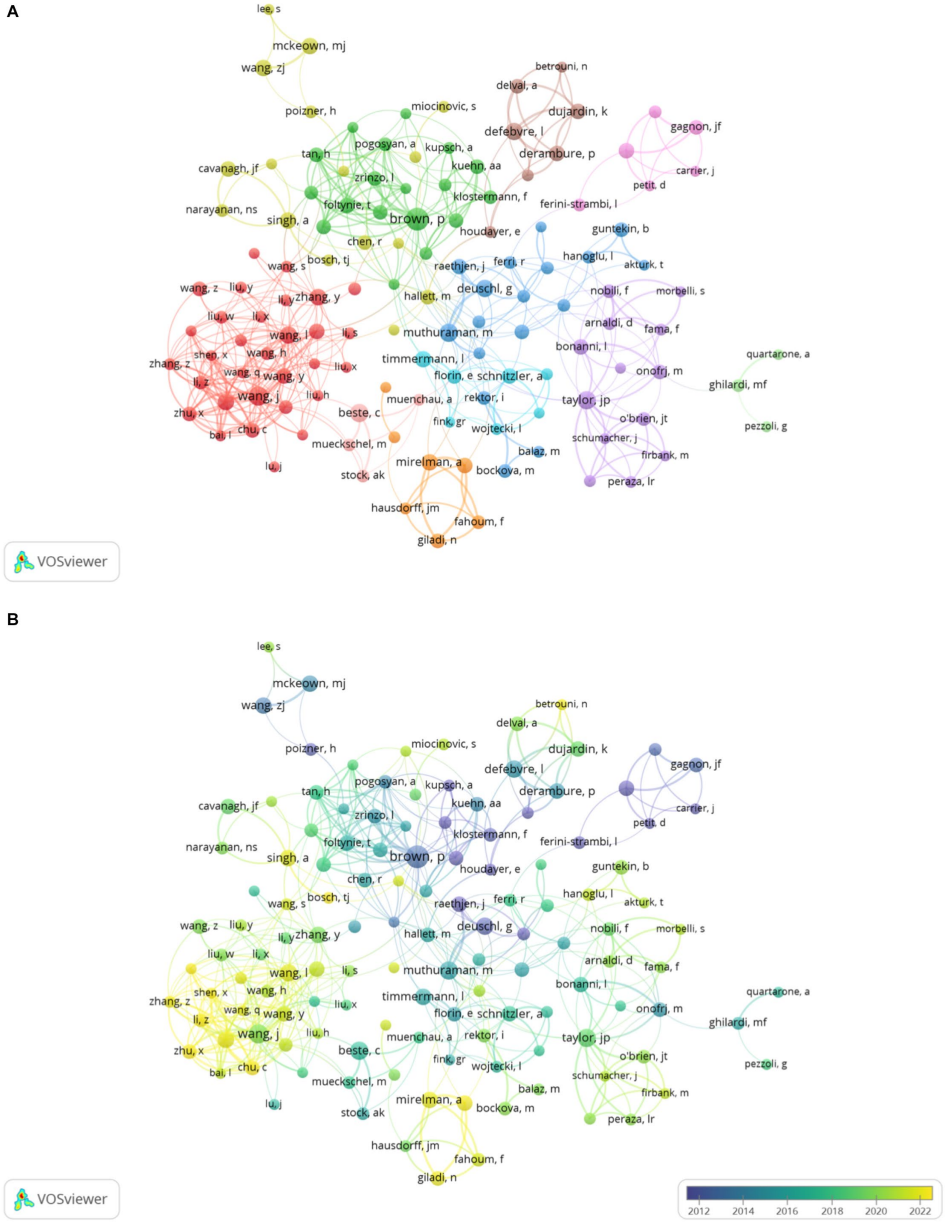
**(A)** Collaborative network knowledge map of author. **(B)** Temporal map of author’s collaborative network.

A total of 6,635 authors have contributed to this research area. Table 3 presents the top 10 authors, who collectively produced 280 publications, representing approximately 17.96% of the total output. Among the most prolific researchers are Brown P, Fuhr P, and Stam C, each of whom contributed 31 publications. Notably, Stam C holds the highest TC and ACPP in this field, while Brown P has the highest h-index and total link strength (TLS), which serves to highlight his substantial influence. Interestingly, although Berendse H is not among the top 5 in publication count, he ranks third for both TC and ACPP, which serves to demonstrate the significant reach and impact of his research. This reflects the profound influence of his work within the scholarly community. Figures 5A,B present a collaborative network and a time overlay graph of authors who have published 6 or more articles. They reveal that collaborative efforts are mainly concentrated among high-impact scholars who work closely within their groups. However, direct collaborations among high-impact scholars are limited.

**Table 3:** Top 10 authors ranked by number of publications.

Rank	Author	Publications (%)	TC	ACPP	H-index	TLS	Location
1	Brown P	31(1.99%)	3,006	96.97	25	82	England
2	Fuhr P	31(1.99%)	692	22.32	18	71	Switzerland
3	Stam CJ	31(1.99%)	3,193	103	21	24	Netherlands
4	Gschwandtner U	30(1.92%)	671	22.37	17	70	Switzerland
5	Taylor JP	26(1.67%)	825	31.73	18	65	England
6	Bonanni L	22(1.41%)	962	43.73	16	29	Italy
7	Güntekin B	21(1.35%)	572	27.24	14	15	Turkey
8	Berendse HW	19(1.22%)	1,271	66.89	15	15	Netherlands
9	Ferri R	19(1.22%)	597	31.42	15	15	Italy
10	Muthuraman M	19(1.22%)	675	35.53	15	30	Hungary

### 3.4 Analysis of journals

**Figure 6:**
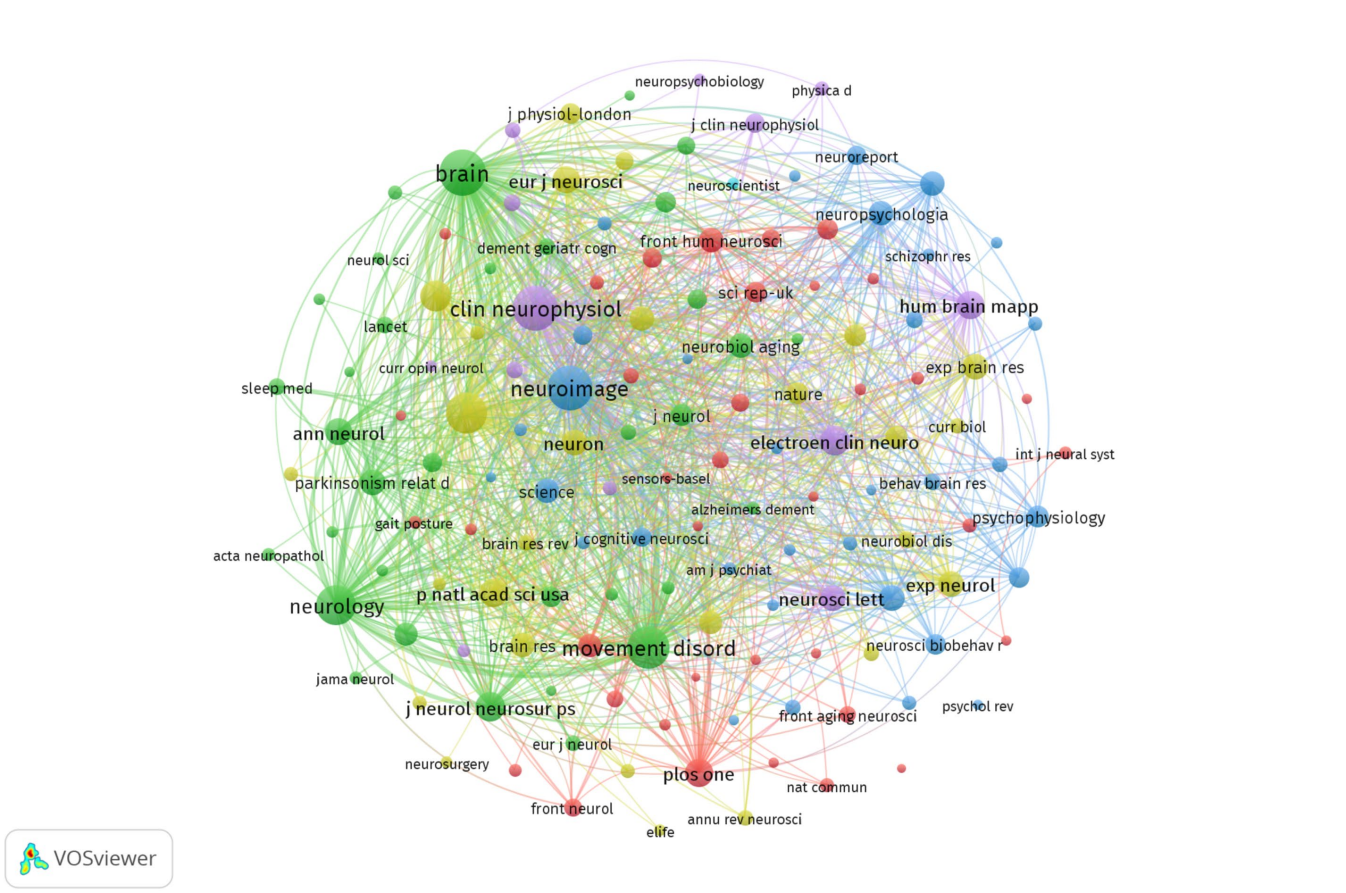
Journal co-citation network knowledge map.

**Figure 7:**
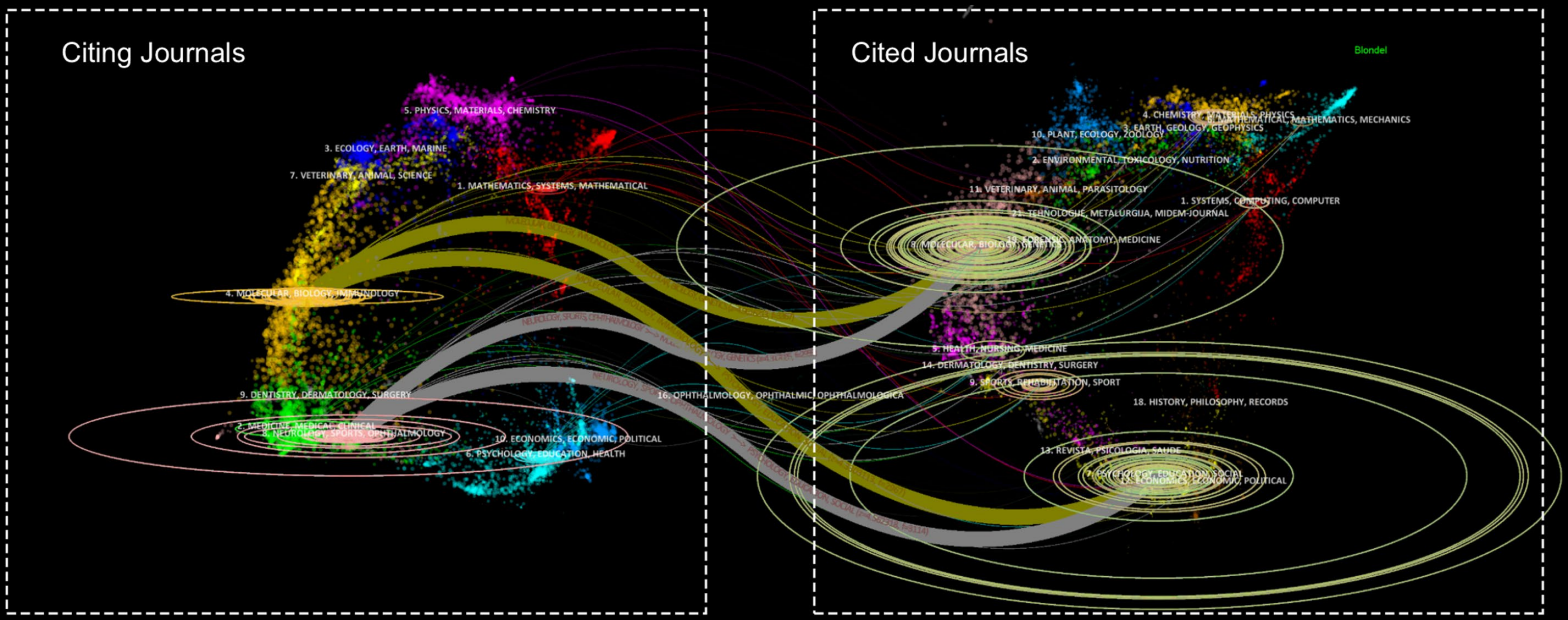
The journal dual-map overlay graph.

The articles included in this study have been published in 390 different academic journals. Table 4 illustrates that the most prolific journal is Clinical Neurophysiology, which has published 95 publications (representing 6.09% of the total), followed by Frontiers in Movement Disorders (52 publications, 3.33%), and Neuroimage (42 publications, 2.69%). The journal with the highest number of publications is Clinical Neurophysiology, while Brain has the highest ACPP, the highest H-index, and the highest impact factor (IF), indicating that it is of a high quality and authoritative in the field of study. All of the journals are classified as either Q1 or Q2, which indicates that they are of a high caliber and have a significant academic impact. Figure 6 depicts the co-citation relationships among the journals. Brain leads with the highest TC and TLS, followed by Neuroimage and Clinical Neurophysiology, as shown in Table 5. Figure 7 depicts the citation relationships among the journals. The journals on the right are cited by those on the left (Guo et al., 2023; He et al., 2023; Li F. et al., 2023). The citation relationships are indicated by colored paths between the citing and cited journals. A two-color primary citation pathway was identified through mapping. This indicates that research published in journals within the field of molecular biology and genetics is primarily cited by research published in molecular biology and immunology. Furthermore, research published in psychology, education, and social sciences is primarily cited by studies published in neurology, sports, and ophthalmology journals.

**Table 4:** Top 10 journals ranked by number of publications.

Rank	Journal	Publications	Citations	ACPP	TLS	IF (2022)	Quartile in category
1	Clinical Neurophysiology	95	4,041	42.54	831	4.7	Q1
2	Movement Disorders	52	2,095	40.29	263	8.6	Q1
3	Neuroimage	42	2,237	53.26	319	5.7	Q1
4	Frontiers in Neurology	40	548	13.7	203	3.4	Q2
5	Frontiers in Human Neuroscience	37	1,015	27.43	175	2.9	Q2
6	Frontiers in Neuroscience	29	232	8	107	4.3	Q2
7	Brain	27	2,201	81.52	438	14.4	Q1
8	Plos One	27	693	25.67	95	3.7	Q2
9	Neuroimage Clinical	26	579	22.27	296	4.2	Q2
10	Scientific Reports	25	250	10	108	4.6	Q2

**Table 5:** Top 10 co-cited journals in citations.

Co-cited journal	Citations	TLS	IF (2022)	Quartile in category
Brain	3,924	249,866	14.4	Q1
Neuroimage	3,611	227,465	5.7	Q1
Clinical Neurophysiology	3,559	229,447	4.7	Q1
Movement Disorders	3,527	202,845	8.6	Q1
Neurology	3,014	171,955	10.1	Q1
Journal of Neuroscience Research	2,958	195,049	4.2	Q2
Electroencephalography and Clinical Neurophysiology	1,393	81,975	None	None
Journal of Neurophysiology	1,357	96,919	2.5	Q3
Journal of Neurology Neurosurgery and Psychiatry	1,313	83,119	11.8	Q1
Proceedings of The National Academy of Sciences of The United States of America	1,087	71,163	11.1	Q1

### 3.5 Analysis of references

**Figure 8:**
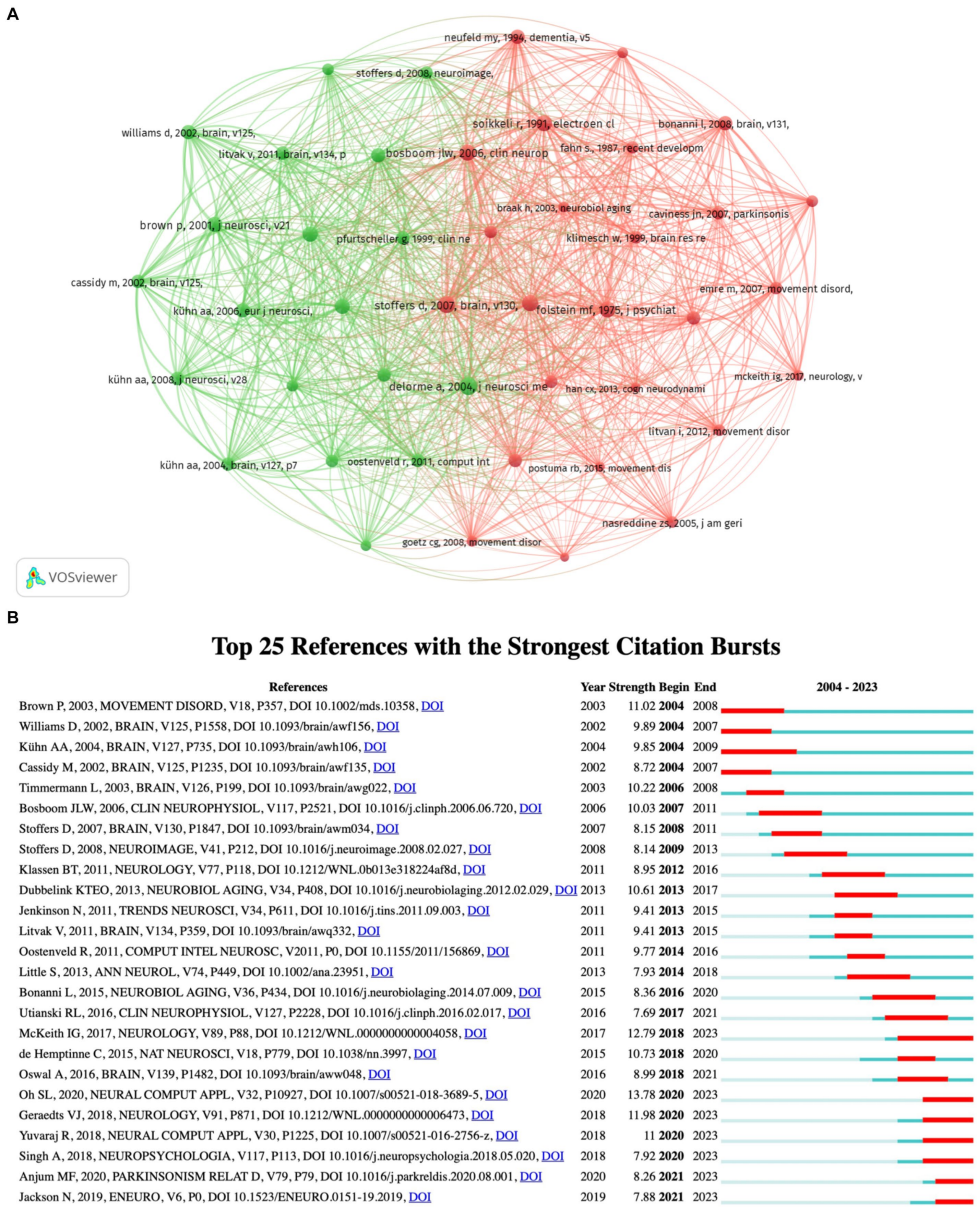
**(A)** Literature co-citation network knowledge map. **(B)** The top 25 references with the highest burst strength.

Figure 8A presents the co-citation networks of publications that have been cited at least 50 times, offering a clear representation of the co-citation relationships among these articles. Table 6 provides a detailed overview of the top 10 most frequently cited publications, which serve to highlight the major research themes in this field. Three of the articles in question delve into software, toolboxes, and the core aspects of EEG processing. Two articles examine the characteristics of disease and the physiological mechanisms associated with PD. Two further articles, authored by Brown P., examine neural oscillations within cortical and basal ganglia circuits in PD. The remaining two articles address topics related to cognition. Citation bursts are defined as references that are cited frequently over time. Identifying these bursts can highlight the most active research areas within a specific time period, based on the subjects of the references (Li Z. et al., 2023). In this study, the timeline is represented by a blue line, with the periods during which citation bursts were investigated marked in red on this line. Figure 8B presents the top 25 articles exhibiting the most pronounced citation bursts. The research with the strongest citation bursts (strength: 13.78) commenced in 2020 and is focused on using a deep learning method to diagnose PD through EEG signals (Oh et al., 2020). It is noteworthy that 7 articles are currently experiencing bursts. These studies in question primarily analyze the spectral characteristics of PD with a view to supporting the diagnostic process.

**Table 6:** Top 10 co-cited references in citations.

Rank	Title	Years	Author	Citations	TLS	Type
1	EEGLAB: an open source toolbox for analysis of single-trial EEG dynamics including independent component analysis	2004	Delorme A	231	606	Article
2	“Mini-mental state.” A practical method for grading the cognitive state of patients for the clinician	1975	Folstein MF	119	477	Editorial Material
3	Parkinsonism: onset, progression, and mortality	1967	Hoehn MM	115	453	Editorial Material
4	Slowing of EEG in Parkinson’s disease	1991	Soikkeli R	106	471	Article
5	Oscillatory Nature of Human Basal Ganglia Activity: Relationship to the Pathophysiology of Parkinson’s Disease	2003	Brown P	99	391	Article
6	Pathological synchronization in Parkinson’s disease: networks, models and treatments	2007	Hammond C	97	406	Review
7	Event-related EEG/MEG synchronization and desynchronization: basic principles	1999	Pfurtscheller G	97	308	Review
8	Slowing of oscillatory brain activity is a stable characteristic of Parkinson’s disease without dementia	2007	Sotoffers D	97	530	Article
9	FieldTrip: Open Source Software for Advanced Analysis of MEG, EEG, and Invasive Electrophysiological Data	2011	Oostenveld R	94	318	Article
10	Dopamine Dependency of Oscillations between Subthalamic Nucleus and Pallidum in Parkinson’s Disease	2001	Brown P	88	400	Article

### 3.6 Analysis of keywords

**Figure 9:**
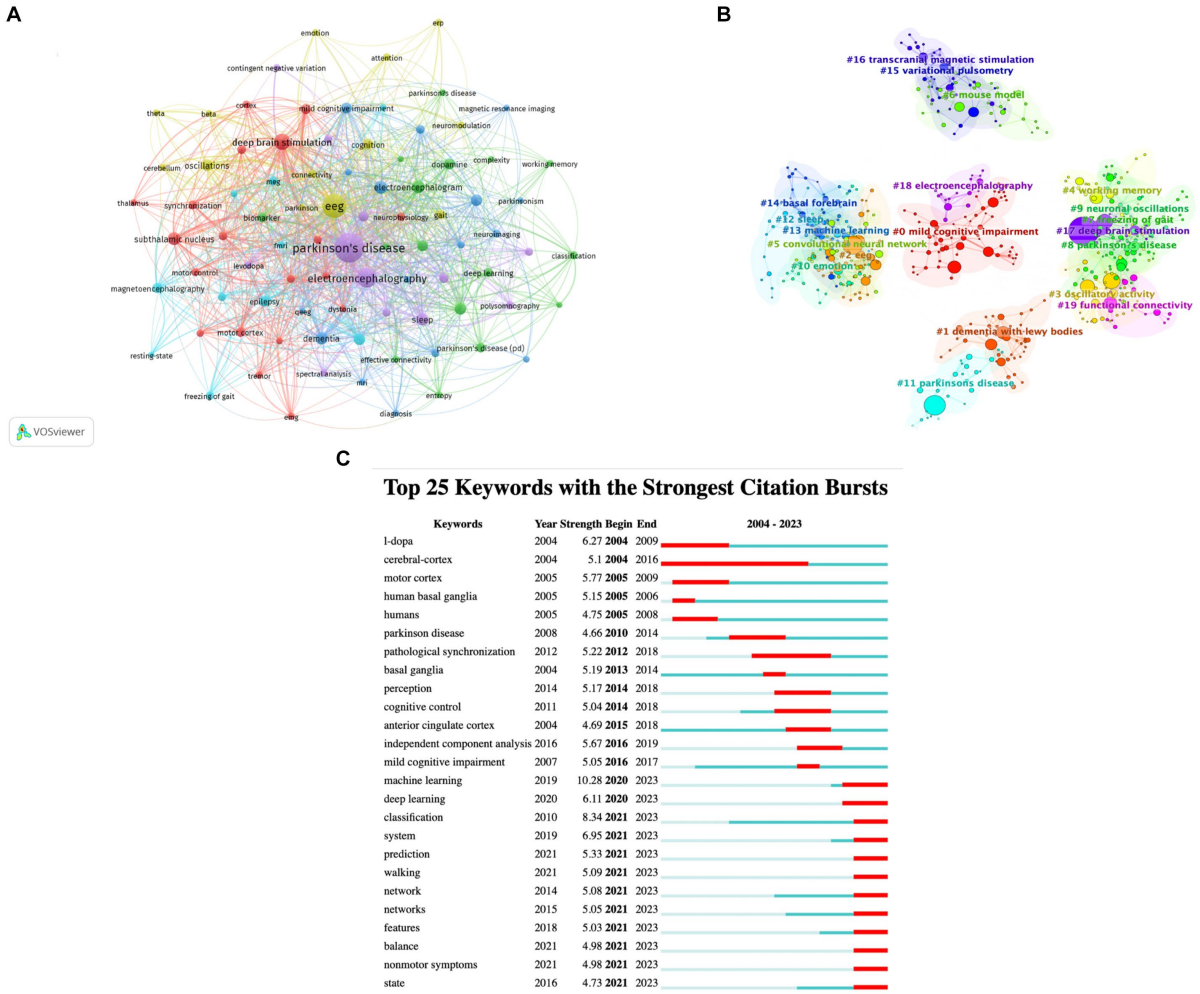
**(A)** Keyword co-occurrence network knowledge map. **(B)** Cluster map of keywords. **(C)** The top 25 keywords with the highest burst strength.

Keywords are concise summaries of the research content of an article. The analysis of high-frequency keywords can reveal the main research topics in a given field. Figure 9A presents the co-occurrence relationships among keywords with at least 10 occurrences. The node size is directly proportional to the frequency of occurrence, and the thickness of the connecting lines correlates with how often the keywords co-occurrence at both ends. Different color clusters reflect the cooperative relationships among keywords. Table 7 lists the top 40 high-frequency keywords and their TLS, providing insights into the primary research topics and emerging trends in this field. Figure 9B presents a visualization cluster map of co-cited references created with CiteSpace. The modularity Q value of 0.7504 and the mean silhouette value of 0.8789 provide evidence of the clustering’s validity, as both values exceed 0.5(Tao et al., 2023). Each circle represents a keyword, and circles of the same color group together around a shared theme, revealing 24 representative clusters. By setting the duration to 1 year, the top 25 keywords with the highest burst intensity were identified and shown in Figure 9C. The keyword “L-dopa” appeared first, “cerebral-cortex” had the longest burst, and keywords like “machine learning,” “deep learning,” “prediction,” and “nonmotor symptoms” are still experiencing bursts. Analyzing these bursts helps identify research hotspots and trends for future development in this field. Particularly, keywords with ongoing bursts offer valuable insights and guidance for further research.

**Table 7:** Top 40 keywords with frequency.

Rank	Keyword	Frequency	TLS	Rank	Keyword	Frequency	TLS
1	Parkinson’s disease	457	813	21	Magnetoencephalography	32	63
2	Eeg	280	511	22	Parkinson’s disease(pd)	27	32
3	Electroencephalography	164	284	23	Quantitative eeg	27	58
4	Deep brain stimulation	107	218	24	Biomarker	26	63
5	Electroencephalogram	56	97	25	Motor cortex	26	50
6	Alzheimers disease	55	104	26	Coherence	25	64
7	Subthalamic nucleus	55	128	27	Gait	24	47
8	Dementia	51	118	28	Synchronization	24	53
9	Sleep	48	104	29	Deep learning	23	40
10	Functional connectivity	47	110	30	Local field potentials	23	46
11	Oscillations	46	118	31	Rem Sleep behavior disorder	22	46
12	Dementia with lewy bodies	45	77	32	Meg	21	66
13	Machine learning	41	97	33	Transcranial magnetic stimulation	20	40
14	Dopamine	40	62	34	Polysomnography	20	41
15	Electroencephalography(eeg)	40	40	35	Electrophysiology	19	40
16	Mild cognitive impairment	38	91	36	Classification	19	38
17	Epilepsy	36	57	37	Neurodegeneration	19	34
18	Basal ganglia	35	76	38	Cognitive impairment	19	21
19	Parkinson disease	35	64	39	Event-related potentials	19	40
20	Cognition	34	84	40	Graph theory	18	37

## 4 Discussion

The landscape of EEG research in PD has experienced a notable surge in academic output from 2004 to 2023. This surge is marked by a notable rise in publications since 2020. The sustained annual publication of over 100 articles during 2021–2023 reflects the growing and dynamic interest in this field. This trend may be attributed to the increasing recognition of PD’s complexity and the potential of EEG as a valuable tool for diagnosis and monitoring, which has spurred extensive research efforts.

In the study of PD through EEG research, certain countries have emerged as early and influential contributors. Germany, the Netherlands, and Canada not only have an earlier average publication year but were also instrumental during the early stages of this field, providing a strong foundation for ongoing studies. The United States, England, and Germany show high centrality and h-index scores, demonstrating their significant roles within the global research community and their contribution to fostering academic partnerships. Notably, these three countries are at the heart of major international collaborative networks. The United States leads an extensive network, engaging with countries such as India, Singapore, Japan, and Israel. Similarly, England spearheads another influential network, partnering with Australia, Italy, the Netherlands, France, and Turkey. Germany plays a pivotal role in a strong collaborative framework involving Denmark, Brazil, Greece, and Hungary. These networks exemplify strategic international alliances that significantly contribute to global research initiatives. Despite ranking sixth in the total number of publications, the Netherlands has the highest ACPP, indicating the broad recognition and impact of its research. This suggests that academic achievements from the Netherlands are frequently cited and highly valued in the scholarly community. Following 2020, China’s research output has rapidly increased, positioning it as one of the largest publishing countries. However, due to its relatively late entry into the field, its global impact remains lower compared to other leading nations. This discrepancy underscores the importance of balancing research quantity with quality and global impact. The 10 countries with the highest number of publications in this field are primarily located in Europe, with two of them in North America. This distribution reflects the prominence of these countries in this field. The extent of research into PD at the national or regional level does not appear to be correlated with the prevalence of PD in a given region (Dammertz et al., 2023; Zheng et al., 2023). This suggests that economic, technological, and other factors exert a more pronounced influence on research productivity.

The University of London is the most prolific institution in the field, with the highest TC and H-index, and has made a significant impact on this field. One of their highly cited research articles investigated the impact of inter-regional cortical synchronization on PD pathophysiology and assessed how dopaminergic therapy and subthalamic nucleus (STN) high-frequency stimulation influence these dynamics. EEG recordings from patients with PD demonstrated a relationship between the 10–35 Hz coherence range and the severity of the disease. Both L-dopa and STN stimulation were found to reduce this cortical coupling, which corresponded with clinical improvements. This implies that these treatments may help restore normal cortical interactions, potentially explaining their similar therapeutic effects in PD management (Silberstein et al., 2005). After the University of London, the University of California is the second-most prolific institution in this field. One of their highly cited articles investigated the deep brain stimulation (DBS) on response inhibition in the STN of PD patients. Using scalp EEG, the researchers observed an increase in beta-band power in a stopping task when the STN was stimulated. Patients with DBS exhibited a faster response time when stimulation was active compared to when it was turned off. This effect was more pronounced in the right frontal cortex, suggesting enhanced messaging within the frontal-basal ganglia circuit (Swann et al., 2011). The number of publications produced by Vrije University Amsterdam is not particularly high, but they are ranked first in the number of citations to their articles. This reflects the high quality and wide recognition of their academic output, which holds significant academic influence in this field. In a previous published review article, they discussed how the brain communication organization is a complex network under normal conditions and how it deteriorates in neurological diseases. The review underscores the significance of contemporary network theory and sophisticated imaging techniques, such as high-density EEG and magnetoencephalography (MEG), in elucidating the intricacies of these networks. The healthy brain is characterized by the formation of “small-world networks” with optimal local and long-distance connectivity, which are vital for cognitive and intellectual functions (Stam and van Straaten, 2012). It is evident that the number of publications does not always correlate with citation metrics or the spread of research influence. For instance, some institutions with fewer publications have exhibited a high ACPP, which indicates the profound reach and recognition of their research. This suggests that while quantity is a factor, the quality and impact of research are of significant importance in determining an institution’s standing in the global research community.

Professor Brown P of Oxford University has the greatest number of published articles, H-index, and TLS, and has a significant impact on this field. His team conducted a series of studies elucidating the role of abnormal brain rhythms, particularly beta oscillations, in the basal ganglia circuits of PD patients. These abnormal oscillations are associated with the rigidity and bradykinesia characteristic of PD (Mallet et al., 2008; Litvak et al., 2011; Little et al., 2012). Furthermore, these oscillations can be influenced by treatments such as DBS and medication (Fischer et al., 2019; Muthuraman et al., 2020). They have employed EEG extensively to study these oscillatory activities, thereby providing crucial insights into their role in PD pathology and treatment efficacy. Professor Stam C from Vrije University Amsterdam is also a high-impact scholar. He is not only one of the most prolific authors in this field of PD research, but also the author with the highest TC and ACPP. His contributions have revolved around elucidating the complex patterns of brain activity associated with PD and exploring the potential for EEG as a tool in the diagnosis and understanding of the disease. He has significantly impacted the field of PD research by employing EEG analysis through the lens of nonlinear dynamical systems (Stam, 2005; Stam and van Straaten, 2012). These studies are of critical importance for the development of more effective diagnostic tools and targeted treatment strategies for PD. EEG-based biomarkers have the potential to be utilized for early diagnosis and for monitoring disease progression. It is noteworthy that a team from Tianjin University, including Zhu X, Chun C, and Liu C, among others, has been active in recent years. In their latest study, they explored the temporal and spectral variations in EEG as manifestations of cognitive impairment. By recruiting early PD patients and conducting microstate analysis in specific frequency bands identified through deep learning, they identify distinctive EEG patterns correlated with cognitive assessment scales (Liu et al., 2023). This study uncovers abnormal microstate characteristics in early PD with mild cognitive impairment (MCI) and offers potential electrophysiological markers for the early recognition of MCI in PD.

An examination of the number of journal publications and their co-citations can provide valuable insights that can assist with the selection of appropriate journals for manuscript submission. The investigation into the EEG research of PD is well-represented across a range of journals, with a concentrated core of journals that are particularly influential in this field. Among the top 10 journals in this field, Clinical Neurophysiology, Movement Disorders, and Neuroimaging are the three most prolific. These three journals have high TC, TLS and IF, with each classified as Q1. It is therefore recommended that new articles be submitted to these journals. In contrast, Brain has the highest IF, TC, and TLS among co-cited journals, functioning as central nodes in the network of scholarly communication. It is closely followed by other highly-cited journals such as Neuroimaging, Clinical Neurophysiology, Movement Disorders, and Neurology. These journals serve not only as platforms for disseminating new discoveries but also as cornerstones in constructing the academic narrative within the field. While the number of publications is a metric that can be used to assess the influence of a journal, co-citation data provides a more accurate reflection of the dissemination and recognition of research within and beyond the field. Furthermore, it is noteworthy that the annual publication of Frontiers in Neurology has experienced a notable surge, commencing in 2019. In a broader context, this bibliometric analysis informs researchers of the most appropriate journals in which to submit their manuscripts for potential publication and serves as a guide for those seeking to keep abreast of leading-edge developments. It is of the utmost importance for researchers to contribute novel findings and engage with the broader academic community through these influential platforms in order to ensure the dissemination and impact of their work.

The significance of an article within a research domain is often determined by the frequency of its citations. The top 10 cited publications included two reviews, six research articles and two editorials. These can be classified into distinct research directions, with EEG studies forming a primary category. Among these, a highly cited article by (Delorme and Makeig, 2004) introduced EEGLAB, an open-source MATLAB toolbox tailored for analyzing single-trial EEG dynamics, inclusive of independent component analysis. EEGLAB is a comprehensive tool for EEG data processing that offers robust capabilities for data importation, visualization, preprocessing, and analysis. It is accessible to both newcomers and seasoned experts in the field. In addition, a noteworthy publication by (Oostenveld et al., 2011) describes FieldTrip, another open-source MATLAB toolbox designed for the analysis of MEG, EEG, and invasive electrophysiological data. FieldTrip encompasses a range of functionalities, from preprocessing to statistical analysis, thereby supporting a diverse spectrum of neuroelectrophysiological research. These software tools have significantly advanced the field of brain neuroscience, catalysing the progress of research by enabling the detailed examination of complex electrophysiological data. The second category delves into the physiological and pathological underpinnings of PD. Hoehn and Yahr (1967) compiled data on 802 patients observed from 1949 to 1964, aiming to understand the variability and progression of the disease across a large group (Brown et al., 2001; Brown, 2003) examining the impact of dopaminergic modulation and motor tasks on neuronal discharge frequency and synchronization within the PD-affected circuits. The researchers identified specific oscillation frequencies, particularly in the beta-band and gamma-band, that are associated with motor functions. This suggests that these oscillations may have functional significance in the pathology and treatment of PD. Moreover, the researchers discovered that levodopa treatment reduced low-frequency activity while enhancing higher-frequency synchronization, indicating that dopamine plays an intricate role in regulating neural activity within the basal ganglia. The third category of research focuses on the application of EEG in the field of PD research. One study by Soikkeli et al. (1991) investigated mid-frontal theta activity during cognitive control tasks in PD patients and found a reduced modulation compared to healthy controls. The researchers examined EEG abnormalities in PD patients, with a focus on the differences between those with and without dementia. A general slowing of EEG signals was observed in PD patients, with the most marked changes occurring in those with dementia, as evidenced by increased delta and theta activity. This distinction in EEG patterns between demented and non-demented PD patients highlights the potential of EEG as a diagnostic tool for detecting neurological changes tied to cognitive decline in PD.

The keyword is the authors’ refining and summarizing of the content of the article, which can reflect the core content of the article. The cluster analysis of keywords identifies the principal research topics in this field, including mild cognitive impairment, dementia with Lewy bodies, EEG, oscillatory activity, working memory, convolutional neural network, mouse model, freezing of gait, Parkinson’s disease, neuronal oscillations, emotion, sleep, machine learning, basal forebrain, variational pulsometry, transcranial magnetic stimulation, deep brain stimulation, functional connectivity, schizophrenia, antisaccade task, CSF examination, and frontal eye field. Based on this keyword analysis, the main research directions over the last two decades have been identified as: (1) Neural oscillations and connectivity: Studies have focused on measuring changes in specific EEG frequency bands, such as beta and gamma oscillations, which are believed to play a key role in the pathophysiology of PD. Many research studies have analyzed how these oscillations correlate with disease severity and response to treatment, providing insights into the underlying neural mechanisms of the disease (Stein and Bar-Gad, 2013; Guan et al., 2022). (2) Innovations in EEG Analysis: Techniques such as graph signal processing and graph convolutional networks have been developed with the objective of enhancing the interpretability and accuracy of EEG analysis (Betrouni et al., 2019). These innovative models integrate local and global network information to enhance the accuracy of PD diagnosis, offering high classification accuracy and detailed visualizations of EEG patterns linked to PD-specific impairments, such as speech disorders. Furthermore, other advanced machine learning techniques, including support vector machines and k-nearest neighbors, are employed to analyze resting-state EEG for cognitive profiling in PD, demonstrating the potential to identify levels of cognitive impairment (Zhao et al., 2024). (3) Impact of therapies on EEG: Research in this field assesses the impact of various therapeutic interventions on EEG dynamics, offering insights into the effects of treatment on brain function. Studies investigate the impact of medications such as levodopa and therapies like DBS on EEG patterns, with the aim of elucidating their mechanisms and optimizing treatment strategies (Tinkhauser et al., 2017; Lofredi et al., 2018; Miocinovic et al., 2018). (4) Cognitive and emotional assessments. Cozac et al. (2016) have demonstrated that quantitative EEG (QEEG) is an effective method for predicting cognitive decline and the risk of dementia in patients with PD. QEEG measures, such as background rhythm frequency and power in specific frequency bands, demonstrate a correlation with neuropsychological assessments and are capable of predicting the onset of dementia. Furthermore, EEG-based emotion classification research is advancing our understanding of how PD affects emotional recognition and expression, utilizing neural network architectures to improve the accuracy and robustness of emotion detection (Klassen et al., 2011). The identification of keyword bursts can reveal changes in the research hotspots and future development trends in this field. In recent years, the identification of burst keywords in this field has revealed a number of emerging research topics, including machine learning, deep learning, prediction, task analysis, walking, network, features, balance, and nonmotor symptoms. These research hotspots not only reflect the current development trends but also provide valuable insights for future research. The emergence of burst keywords such as machine learning and deep learning indicates the integration of advanced computational methods in PD research. In particular, these methods have been employed to enhance predictive models and to facilitate the understanding of complex datasets (Geraedts et al., 2021; Aljalal et al., 2022; Parajuli et al., 2023; Diao et al., 2024; Riasi et al., 2024). Other keywords, such as “prediction” and “task analysis,” reflect a growing focus on developing tools that can anticipate disease progression and evaluate patient responses to various tasks (Bar-On et al., 2023; Memon et al., 2023; Huang et al., 2024). These tools are designed to inform the development of personalized treatment strategies. The growing interest in “walking,” “balance,” and “nonmotor symptoms (Conti et al., 2023)” reflects a broader approach to understanding PD, emphasizing the need to address the multifaceted nature of the disease that affects both motor and cognitive functions (Bosch et al., 2021; Costa et al., 2023; Guo et al., 2024). As researchers continue to investigate these topics, integrating advanced technologies and holistic approaches promises to drive innovations in the management of PD, potentially leading to earlier detection, more effective treatments, and better patient outcomes. This convergence of technology and clinical practice paves the way for transformative advancements in the care and study of PD.

## 5 Limitation

Firstly, this bibliometric analysis is confined to literature indexed from the SCI-Expanded within the WoSCC, which may result in the overlooking of significant contributions from other databases or journals not covered by this collection. The primary reason for this is the utilization of an online bibliometric visualization and analysis website, which currently only supports the WoS database. Furthermore, the focus on English-language publications may exclude valuable research published in other languages, which could offer different insights or corroborate current findings. This language bias could limit the comprehensiveness of our understanding of the global research landscape in the field. In the future, translation tools or enhanced national communication may be employed to include articles in a greater number of languages. Moreover, by concentrating primarily on peer-reviewed articles and reviews, other potentially impactful works such as conference papers, dissertations, or technical reports might have been excluded, which could contribute innovative approaches or preliminary findings not yet available in journal articles. Additionally, the inherent limitations of bibliometric analysis, such as the dependence on keyword frequency and co-citation networks, might not fully capture the nuanced interrelations or emerging trends that qualitative assessments could reveal. Finally, this article includes studies from 2004 to 2023, which inevitably limits the inclusion of earlier as well as more recent studies. In conclusion, while this study provides a structured overview of EEG research in PD using bibliometric methods, it is important to interpret its findings within the context of these outlined limitations. Future research should aim to expand the scope of databases, include multilingual publications, and incorporate a broader range of document types to enhance the depth and accuracy of the portrayal of the research landscape.

## 6 Conclusion

From 2004 to 2023, EEG research in PD has shown a significant increase in both publication and citation counts. This reflects the growing interest and significant progress in this field. The application of bibliometrics and visualization methods has provided insights into the evolution, impact, and future direction of this area of research. This study demonstrates that countries such as the United States, Germany, and England are at the forefront of EEG research and innovation, contributing a substantial volume of research and technological advancement. The extensive collaboration between scholars and institutions around the world serves to illustrate the collective global effort to address the complexities of PD through EEG research. The identification of key research institutions, influential scholars, and significant publications illustrates the dynamic and interconnected nature of the field. Notably, the integration of EEG technology has revealed the complex neurological underpinnings of PD, providing innovative diagnostic tools and therapeutic insights. In the future, this field will continue to adopt advanced EEG technologies and machine learning models in order to deepen our understanding of the disease and improve treatments.

## Data availability statement

The original contributions presented in the study are included in the article/Supplementary material, further inquiries can be directed to the corresponding authors.

## Author contributions

X-YL: Conceptualization, Data curation, Formal analysis, Methodology, Visualization, Writing – original draft, Writing – review & editing. Y-XG: Investigation, Methodology, Writing – review & editing. T-TQ: Validation, Writing – review & editing. L-HZ: Visualization, Writing – review & editing. L-QL: Resources, Writing – review & editing. YG: Funding acquisition, Writing – review & editing. T-FY: Conceptualization, Writing – review & editing.
